# Injectable Magnetic Hydrogel Incorporated with Anti‐Inflammatory Peptide for Efficient Magnetothermal Treatment of Endometriosis

**DOI:** 10.1002/advs.202409778

**Published:** 2024-10-07

**Authors:** Huaichao Liu, Xiaohui Dai, Na Li, Le Zhang, Zihan Wang, Ke Ren, Yulei Li, Xiao Sun, Jipeng Wan

**Affiliations:** ^1^ Department of Gynecology Ji Nan Key Laboratory of Diagnosis and Treatment of Major Gynaecological Disease Shandong Provincial Hospital Affiliated to Shandong First Medical University Jinan 250021 China; ^2^ School of Chemistry and Pharmaceutical Engineering Medical Science and Technology Innovation Center Shandong First Medical University and Shandong Academy of Medical Sciences Jinan 250000 China

**Keywords:** anti‐inflammatory therapy, drug release, endometriosis, injectable hydrogel, magnetothermal treatment

## Abstract

Endometriosis is a prevalent gynecological condition characterized by chronic pelvic pain, dysmenorrhea, and infertility, affecting ≈176 million women of reproductive age worldwide. Current treatments, including pharmacological and surgical interventions, are often associated with significant side effects and high recurrence rates. Consequently, there is an urgent need for innovative and safer therapeutic approaches. In this study, an injectable magnetic hydrogel nanosystem is developed designed for the dual‐purpose magnetothermal and anti‐inflammatory treatment of endometriosis. This hydrogel incorporates Fe_3_O_4_ nanoparticles alongside an anti‐inflammatory peptide. Upon magnetic activation, the Fe_3_O_4_ nanoparticles induce a localized hyperthermic response, raising the temperature of endometriotic lesions to 63.3 °C, effectively destroying endometriotic cells. Concurrently, the thermally responsive hydrogel facilitates the controlled release of the anti‐inflammatory peptide, thus modulating the inflammatory milieu. The biocompatibility and complete in vivo degradability of the hydrogel further enhance its therapeutic potential. The in vivo studies demonstrated that this injectable magnetic hydrogel system achieved a 90% reduction in the volume of endometriotic lesions and significantly decreased inflammatory markers, offering a promising non‐invasive treatment modality for endometriosis. By integrating precise lesion ablation with the modulation of the inflammatory microenvironment, this system represents a novel approach to the clinical management of endometriosis.

## Graphical abstract

Here, an injectable magnetic hydrogel nanosystem for the dual‐purpose magnetothermal and anti‐inflammatory treatment of endometriosis is well designed. Upon magnetic activation, the hydrogel containing Fe_3_O_4_ nanoparticles can induce a localized hyperthermic response, effectively destroying endometriotic cells and facilitating the release of anti‐inflammatory peptide. Consequently, this work offers a promising non‐invasive treatment modality for synergistical magnetothermal and anti‐inflammatory treatment of endometriosis.

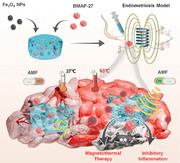

## 1 Introduction

Endometriosis is a prevalent gynecological condition characterized by the ectopic growth of endometrial‐like tissue outside the uterine cavity, which leads to severe pelvic pain and substantially impairs the quality of life.^[^ ^1^ ^]^ It is estimated that 10% to 15% of women of reproductive age are affected by endometriosis, with infertility observed in over 40% of these individuals.^[^ ^2^ ^]^ A critical component of the pathophysiology of endometriosis is chronic inflammation, mediated by the overproduction of proinflammatory cytokines.^[^ ^1^, ^3^ ^]^ Current therapeutic interventions, including surgical resection and pharmacological treatments, are associated with considerable side effects and high recurrence rates. Given that inflammation not only facilitates lesion proliferation but also intensifies pain and contributes to the persistence of the disease, effective management of the inflammatory microenvironment is crucial.^[^ ^4^ ^]^ Therefore, there is a pressing need for non‐invasive therapeutic strategies that target both lesion ablation and inflammation modulation, thereby improving long‐term outcomes for patients.

Nanoparticle‐based platforms have demonstrated substantial potential in the treatment of cancer and inflammatory conditions, owing to their capabilities in drug delivery and bio‐imaging.^[^ ^5^ ^]^ Notably, nanoparticle‐mediated magnetic hyperthermia has emerged as a promising therapeutic approach, leveraging localized temperature increases to enhance treatment efficacy.^[^ ^6^ ^]^ Magnetic nanoparticles are favored for their cost‐effectiveness, high stability, and superior tissue penetration properties.^[^ ^5^, ^7^ ^]^ For instance, Chen demonstrated that hollow Fe_3_O_4_ crystals, when subjected to an alternating magnetic field, significantly elevate local temperatures within tumors, effectively inhibiting tumor growth while minimizing thermal damage to adjacent tissues.^[^ ^8^ ^]^ Given the pathophysiological similarities between solid tumors and endometriosis, such as abnormal tissue proliferation and angiogenesis, this technology has also been explored for endometriosis treatment.^[^ ^9^ ^]^ Magnetic nanoparticles offer a non‐invasive hyperthermia approach for the localized ablation of endometrial lesions.^[^ ^10^ ^]^ However, the rapid clearance of these particles by the reticuloendothelial system poses a challenge, necessitating advancements in delivery and drug release strategies.

In this context, hydrogels have emerged as a highly promising biomaterial within the medical field, particularly for drug delivery applications. These systems enable the localized administration of high drug concentrations directly to pathological sites and can facilitate drug release in response to various stimuli, including pH, temperature, magnetic fields, and light.^[^ ^11^ ^]^ Direct intratumoral injection of hydrogels has proven to be an effective drug delivery strategy.^[^ ^12^ ^]^ Thermosensitive hydrogel systems, which form stable gel structures upon injection, allow for sustained drug release through controlled melting of the hydrogel, thereby extending therapeutic effects and reducing dosing frequency.^[^ ^13^ ^]^ Compared with traditional oral or intravenous administration routes, local hydrogel‐based treatments offer reduced systemic toxicity and enhanced drug efficacy.^[^ ^14^ ^]^ For example, Cai et al. developed a temperature‐sensitive multifunctional hydrogel patch containing lidocaine, which accelerates wound closure by controlling drug release and providing analgesic, antioxidant, and hemostatic effects.^[^ ^15^ ^]^ Similarly, Cao et al. designed a hybrid hydrogel patch for wound healing, where the photothermal effect of magnetic nanoparticles under near‐infrared irradiation triggers the release of proangiogenic asiatic acid.^[^ ^16^ ^]^ Agarose (AG) hydrogels, known for their excellent biocompatibility and biodegradability, are metabolized and cleared via physiological pathways, minimizing toxic side effects and long‐term harm.^[^ ^17^ ^]^ Therefore, the development of hydrogel‐based systems with controlled response mechanisms represents a promising strategy for the treatment of endometriosis.

**Figure 1:**
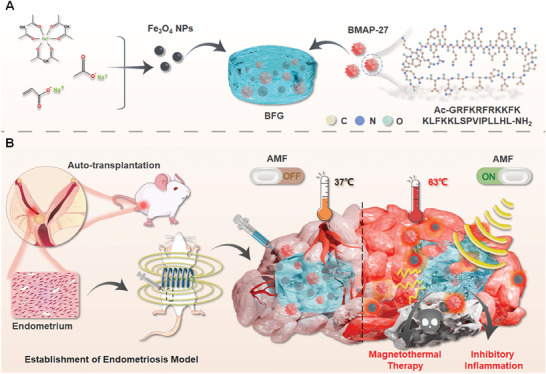
Schematic diagram illustrating the preparation and mechanism of action of injectable magnetic hydrogel (BMAP‐27/Fe_3_O_4_@Gel) for magnetothermal/anti‐inflammatory synergistic therapy of endometriosis: the BMAP‐27/Fe_3_O_4_@Gel composed of Fe_3_O_4_ and BMAP‐27 (Ac‐GRFKRFRKKFKKLFKKLSPVIPLLHL‐NH_2_) was injected into the lesion site of endometriosis. Due to the magnetic activation properties of Fe_3_O_4_, the hydrogel melted and heated the lesion site to 63 °C, resulting in apoptosis and rapid release of BMAP‐27 anti‐inflammatory peptide, which realized the regulation of inflammatory environment in endometriosis and the elimination of endometriosis lesions.

In this study, we developed a novel injectable magnetic hydrogel nanosystem incorporating Fe_3_O_4_ nanoparticles and bovine myeloid antimicrobial peptide 27 (BMAP‐27), which possesses significant anti‐inflammatory and anti‐tumor properties, making it a promising candidate for endometriosis management. This system allows for precise control of drug release via an external alternating magnetic field (AMF) (**Figure** **1**). The hydrogel exhibits several advantageous characteristics, including injectability, biocompatibility, and structural stability. Its injectable nature ensures targeted in situ administration around endometriosis lesions. Upon exposure to an external AMF, the Fe_3_O_4_ nanoparticles induce localized hyperthermia, leading to apoptosis of endometriotic cells and subsequent tissue ablation, thereby providing effective magnetothermal therapy. The extended residence time of the hydrogel enhances the local magnetothermal effect, enabling efficient lesion eradication with a single intervention. Additionally, the magnetothermal‐induced melting of the hydrogel promotes the release of BMAP‐27, which contributes to the modulation of the inflammatory microenvironment within the affected tissue. Consequently, the BMAP‐27/Fe_3_O_4_@Gel hydrogel system offers a non‐toxic, biodegradable therapeutic approach that effectively clears lesion tissue and mitigates inflammation, ultimately enhancing the clinical outcomes for patients with endometriosis.

## 2 Results and Discussion

### 2.1 Preparation and Characterization of Fe_3_O_4_ and BMAP‐27/Fe_3_O_4_@Gel

**Figure 2:**
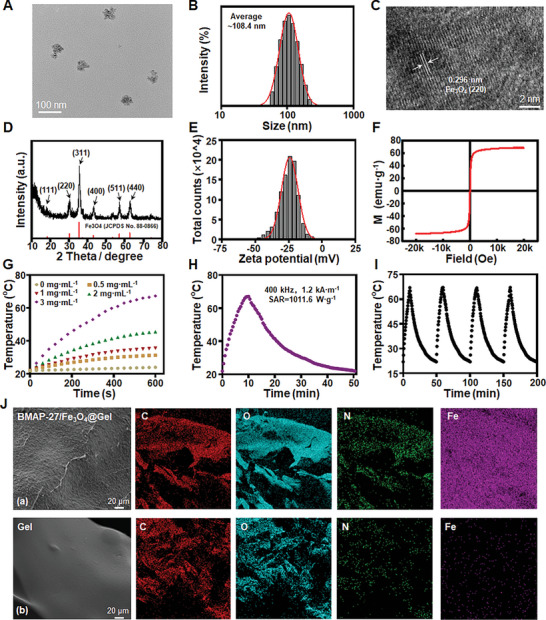
Synthesis and characterization of Fe_3_O_4_ and BMAP‐27/Fe_3_O_4_@Gel nanocomposites. A) TEM image of Fe_3_O_4_. Scale bar = 100 nm. B) DLS of Fe_3_O_4_ in water. C) HR‐TEM of Fe_3_O_4_ NPs. Scale bar = 2 nm. D) XRD spectrum of Fe_3_O_4_. E) Zeta potential of Fe_3_O_4_. F) Hysteresis loop of Fe_3_O_4_. G) Temperature curves of Fe_3_O_4_ under an AMF (400 kHz, 1.2 kA m^−1^). H) SAR of Fe_3_O_4_ (3 mg mL^−1^). I) Magnetothermal stability of Fe_3_O_4_ (3 mg mL^−1^). J) SEM images of BMAP‐27/Fe_3_O_4_@Gel and Gel. Scale bar = 20 µm.

Fe_3_O_4_ nanoparticles were synthesized using the polyol hydrothermal method.^[^ ^18^ ^]^ Briefly, magnetic Fe_3_O_4_ nanoparticles were successfully prepared using ferrous acetylacetonate as the iron precursor, sodium acetate as the alkaline hydrolysis agent, and diethylene glycol as the stabilizer. The particle size of the Fe_3_O_4_ nanoparticles was modulated by varying the ratio of ethylene glycol to diethylene glycol. Transmission electron microscopy (TEM) images revealed that the morphology of Fe_3_O_4_ nanoparticles were spherical and the particle size was ≈70 nm (**Figure** **2**A; Figure S1, Supporting Information). Dynamic light scattering (DLS) measurements indicated hydrodynamic diameters of 108.4 nm in water and 109.3 nm in PBS (pH 7.4), which were larger than the TEM results, attributed to Brownian motion and hydration effects (Figure 2B; Figure S2, Supporting Information). High‐resolution TEM (HRTEM) imaging revealed a lattice spacing of 0.296 nm, consistent with the (220) plane of Fe_3_O_4_ (Figure 2C). X‐ray diffraction (XRD) analysis further corroborated the crystal structure of Fe_3_O_4_, with diffraction peaks aligning with the spinel structure (JCPDS‐88‐0866) (Figure 2D). The zeta potential of Fe_3_O_4_ in water was measured at −25.1 mV (Figure 2E).

The magnetic properties of Fe_3_O_4_ nanoparticles are crucial for magnetothermal therapy applications. Vibrating sample magnetometer analysis demonstrated a saturation magnetization of 68.2 emu g^−1^, with noticeable hysteresis at low magnetic fields (Figure 2F). To evaluate the magnetothermal performance, the temperature of Fe_3_O_4_ under an external AMF (400 kHz, 1.2 kA m^−1^) was monitored. Results indicated a dose‐dependent temperature increase (Figure 2G), and the specific absorption rate (SAR) was determined to be 1011.6 W g^−1^, confirming the nanoparticles’ exceptional magnetothermal properties (Figure 2H). Notably, the temperature of Fe_3_O_4_ (3 mg mL^−1^) reached 67.1 °C within 10 min of magnetic field exposure, sufficient to induce hyperthermia and ablate endometrial lesions. The temperature remained stable across four cycles of AMF activation, indicating excellent magnetothermal stability (Figure 2I). These findings highlight Fe_3_O_4_’s robust magnetothermal and biocompatibility properties, establishing it as a promising candidate for safe and effective magnetothermal therapy. Therefore, these results have intensified our interest in exploring magnetocaloric therapy for various diseases, particularly endometriosis. The temperature‐sensitive hydrogel system can release drugs by melting the hydrogel, which has good biological safety.^[^ ^13a^ ^]^ Consequently, the BMAP‐27/Fe_3_O_4_@Gel composite hydrogel was synthesized by dispersing the BMAP‐27 peptide and Fe_3_O_4_ with magnetothermal properties in AG solution.

The material properties of the BMAP‐27/Fe_3_O_4_@Gel composite hydrogels were systematically characterized. The scanning electron microscope (SEM) images of the BMAP‐27/Fe_3_O_4_@Gel composite hydrogel synthesized by mixing Fe_3_O_4_ nanoparticles with BMAP‐27 peptide at a fixed ratio was shown in Figure 2J, which exhibited some wrinkles. For comparative analysis, Fe_3_O_4_@Gels were synthesized by incorporating Fe_3_O_4_ nanoparticles into an agar hydrogel matrix. Energy dispersive X‐ray spectroscopy (EDS) was employed to quantify the atomic composition of the hydrogels, with and without the incorporation of Fe_3_O_4_ and BMAP‐27 peptide. The EDS results confirmed the presence of nitrogen and iron within the composite hydrogels (Figure 2J; Figure S3, Supporting Information). Fourier transform infrared (FTIR) spectroscopy further corroborated the successful synthesis of BMAP‐27/Fe_3_O_4_@Gel (Figure S4, Supporting Information). As was reported, the characteristic bands of polyacrylic acid (PAA) are observed between 1219 and 1800 cm^−1^, including two strong peeks at 1408 and 1551 cm^−1^, ascribed to the symmetric and asymmetric modes of carboxylate anion (COO^−^),^[^ ^19^ ^]^ respectively. Our synthesized Fe_3_O_4_ nanoparticles showed an apparent carboxyl (−COOH) peak at 1408 cm^−1^, indicating that PAA has functionalized its surface. The peaks observed at 560 cm^−1^ for both Fe_3_O_4_@Gel and BMAP‐27/Fe_3_O_4_@Gel were attributed to the Fe─O bond stretching vibrations in Fe_3_O_4_.^[^ ^20^ ^]^ Additionally, the peak at 1660 cm^−1^ in BMAP‐27/Fe_3_O_4_@Gel was associated with the C═C bond stretching vibrations characteristic of the BMAP‐27 peptide.^[^ ^21^ ^]^ These findings collectively confirmed the successful encapsulation of BMAP‐27 peptide and Fe_3_O_4_ nanoparticles within the composite hydrogel matrix.

### 2.2 Performance Evaluation of BMAP‐27/Fe_3_O_4_@Gel

**Figure 3:**
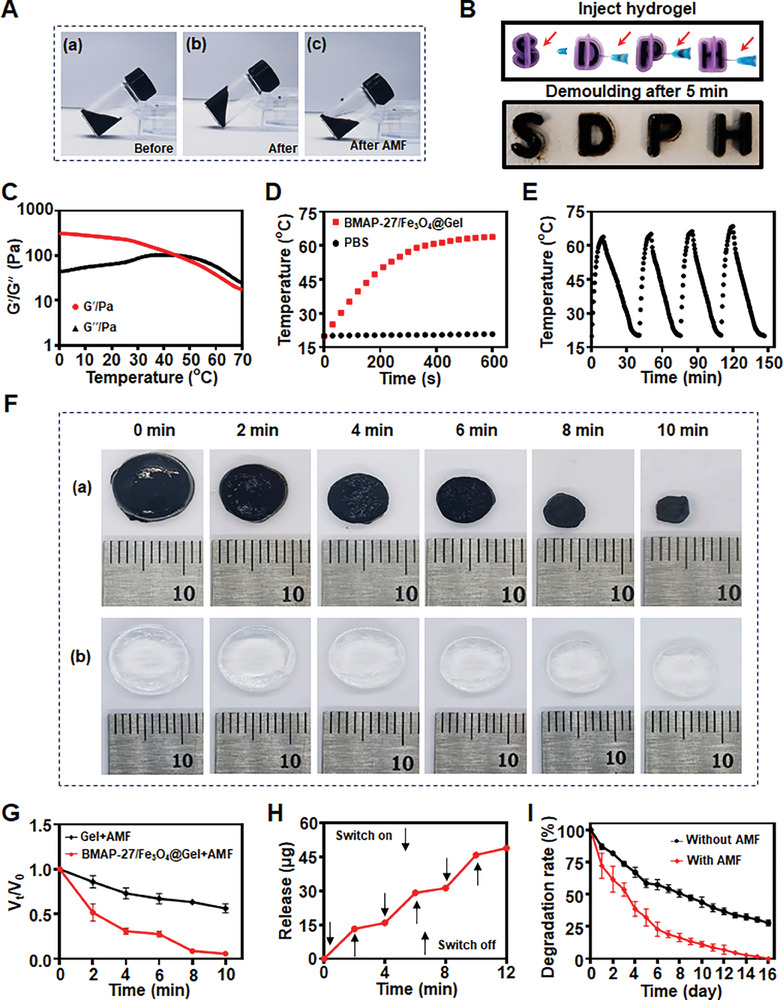
Performance evaluation of BMAP‐27/Fe_3_O_4_@Gel. A) Gelation capability of BMAP‐27/Fe_3_O_4_@Gel. B) Injectability performance of BMAP‐27/Fe_3_O_4_@Gel. C) Storage modulus (G′) and loss modulus (G′′) of BMAP‐27/Fe_3_O_4_@Gel across varying temperatures. D) Temperature curves of BMAP‐27/Fe_3_O_4_@Gel under an external AMF (400 kHz, 1.2 kA m^−1^). E) Magnetic hyperthermia stability of BMAP‐27/Fe_3_O_4_@Gel. F) Contraction response of BMAP‐27/Fe_3_O_4_@Gel compared with Gel under AMF, and G) the corresponding quantitative analysis (*n* = 3). H) Cumulative release profile of BMAP‐27 from BMAP‐27/Fe_3_O_4_@Gel. I) Degradation rate of BMAP‐27/Fe_3_O_4_@Gel with and without AMF exposure (*n* = 3).

To verify the gelation capabilities of BMAP‐27/Fe_3_O_4_@Gel, we first injected the liquid hydrogel into a bottle under flowing conditions. Upon rotating the bottle 180°, the hydrogel remained aggregated on the original side. However, after 5 min of exposure to magnetic radiation, the hydrogel exhibited semi‐flowing behavior and reflowed to the bottom (**Figure** **3**A), demonstrating its responsiveness to magnetic stimulation. The injectability of BMAP‐27/Fe_3_O_4_@Gel was further assessed by measuring the injection pressure.^[^ ^17a^ ^]^ The results showed that the injectability did not significantly differ among the samples, with the injection pressure required for BMAP‐27/Fe_3_O_4_@Gel being ≈10 N (Figure S5 and Table S1, Supporting Information), indicating that manual injection could be performed without the need for auxiliary equipment.^[^ ^17b^ ^]^ Furthermore, when the gel was injected into a mold using a 22 G needle, the formation of the letters “SDPH” was clearly discernible after 5 min, confirming the gel's robust gelation properties (Figure 3B). Subsequently, the rheological behavior of BMAP‐27/Fe_3_O_4_@Gel was characterized using a rheometer, with a particular focus on the effect of temperature on its fluidity. As shown in Figure 3C, the storage modulus (G′) and loss modulus (G′′) were recorded across varying temperatures. The gel maintained a solid‐like state (G′ > G′′) until reaching a critical temperature of 47 °C, beyond which G′′ surpassed G′, indicating a transition to a liquid state with enhanced migratory properties.^[^ ^22^ ^]^ This phase transition facilitated the accelerated release of Fe_3_O_4_ nanoparticles and the BMAP‐27 peptide. Additionally, rheological assessments were conducted using oscillatory frequency sweeps (0.01–10 Hz, 1% strain) and oscillatory strain sweeps (0.01–100% strain, 1 Hz) (Figure S6, Supporting Information). The storage modulus (G') obtained from frequency sweep measurements (0.01–10 Hz, 1% strain) revealed that the incorporation of Fe_3_O_4_ into the hydrogel significantly increased the G′ values, which remained consistent across the oscillatory frequency spectrum (Figure S6A, Supporting Information). Strain sweep results further elucidated the correlation between the deformation behavior of the hydrogels and the Fe_3_O_4_ content in the BMAP‐27/Fe_3_O_4_@Gel, demonstrating that an increase in Fe_3_O_4_ nanoparticles corresponded to enhanced compressive strength and fracture strain (Figure S6B, Supporting Information).

We subsequently evaluated the magnetothermal performance of BMAP‐27/Fe_3_O_4_@Gel to determine its potential efficacy in magnetothermal therapy for endometriosis. Under an AMF (400 kHz, 1.2 kA m^−1^),^[^ ^23^ ^]^ BMAP‐27/Fe_3_O_4_@Gel containing 3 mg mL^−1^ Fe_3_O_4_ nanoparticles exhibited a rapid temperature increase to 63.9 °C within 10 min (Figure 3D; Figure S7, Supporting Information). This increase in temperature facilitated the complete melting of the hydrogel, thereby enabling controlled release of BMAP‐27 and Fe_3_O_4_. To further characterize BMAP‐27/Fe_3_O_4_@Gel, we employed a magnetic heat cycling technique (Figure 3E), in which the hydrogel demonstrated rapid heating under AMF followed by gradual cooling to its baseline temperature. The maximum temperature achieved in subsequent cycles was marginally higher than that of the initial cycle, likely because of the moisture evaporation from the hydrogel, indicative of robust magnetothermal stability. Given the importance of biodegradability for in vivo applications, we observed that BMAP‐27/Fe_3_O_4_@Gel exhibited greater dynamic volume changes and a more pronounced contraction response under AMF compared to the hydrogel alone (Figure 3F,G). The release rate of BMAP‐27 peptide from BMAP‐27/Fe_3_O_4_@Gel was significantly higher upon AMF exposure relative to the control without AMF exposure (Figure 3H; Figure S8, Supporting Information), underscoring the ability of AMF to thermally activate the injectable hydrogel system and regulate the release of both BMAP‐27 peptide and Fe_3_O_4_ NPs. Additionally, the release of Fe_3_O_4_ from BMAP‐27/Fe_3_O_4_@Gel demonstrated a time‐dependent increase (Figure S9, Supporting Information). The hydrogel degraded to 23% of its original mass within 6 days following AMF exposure (Figure 3I), highlighting its remarkable degradation efficiency under magnetic hyperthermia. These findings suggest that BMAP‐27/Fe_3_O_4_@Gel offers promising conditions for the treatment of endometriosis in vivo.

### 2.3 In Vitro Magnetothermal Therapy

**Figure 4:**
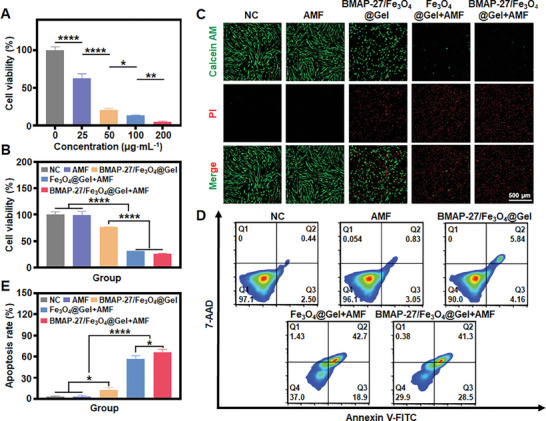
In vitro magnetothermal therapy of BMAP‐27/Fe_3_O_4_@Gel. A) Viability of ESCs treated with varying concentrations of BMAP‐27 peptide (0, 25, 50, 100, or 200 µg mL^−1^) (*n* = 5). B) MTT assay results for ESCs subjected to different treatment conditions. C) Calcein‐AM/PI staining assay demonstrating the effects of various treatments on ESCs (*n* = 5). Scale bar = 500 µm. D) Flow cytometry analysis of apoptotic ESCs following different treatments, with E) corresponding quantitative analysis (*n* = 3). *p < 0.05, **p < 0.01, ****p < 0.0001. Statistical significance was determined using one‐way ANOVA.

The magnetothermal cytotoxicity of BMAP‐27/Fe_3_O_4_@Gel was evaluated using the methyl thiazolyl tetrazolium (MTT) assay with 3T3 and HUVEC cell lines.^[^ ^24^ ^]^ Cell viability remained above 90% when cultured with BMAP‐27/Fe_3_O_4_@Gel, demonstrating good biocompatibility (Figure S10, Supporting Information). Additionally, the direct inhibitory effects of the BMAP‐27 peptide on human endometrial stromal cells (ESCs) were preliminarily examined. As shown in **Figure** **4**A, ESCs treated with the BMAP‐27 peptide exhibited a dose‐dependent reduction in cell viability, indicating a potent growth‐inhibitory effect. However, the encapsulation of the BMAP‐27 peptide (200 µg mL^−1^) within the hydrogel significantly attenuated its inhibitory effect on ESCs. The survival rate in the BMAP‐27/Fe_3_O_4_@Gel group was higher than in the BMAP‐27/Fe_3_O_4_@Gel+AMF group, underscoring the potent magnetothermal cytotoxicity of the hydrogel (Figure 4B). The underlying mechanism of magnetothermal cytotoxicity was further explored using live/dead cell staining. Viable cells were stained with green fluorescence (calcein‐AM), while dead cells were marked with red fluorescence (propidium iodide, PI) (Figure 4C; Figure S11, Supporting Information).^[^ ^17b^ ^]^ The control and AMF groups exhibited bright green fluorescence, indicating no detrimental effect of magnetic radiation on ESCs. In contrast, the Fe_3_O_4_@Gel+AMF and BMAP‐27/Fe_3_O_4_@Gel+AMF groups exhibited a more intense red fluorescence than the BMAP‐27/Fe_3_O_4_@Gel group, indicating that the primary cytotoxic effect was mediated by the AMF‐triggered conversion of Fe_3_O_4_ magnetic energy into thermal energy. The green fluorescence in the BMAP‐27/Fe_3_O_4_@Gel group was diminished compared with the control group, suggesting that the BMAP‐27 peptide's growth inhibition effect effectively overcame treatment limitations, thereby enhancing therapeutic efficacy. These results were corroborated by flow cytometry, which showed an apoptosis rate of 69.8% in the BMAP‐27/Fe_3_O_4_@Gel+AMF group, confirming that magnetothermal therapy induced apoptosis and significantly decreased cell viability in endometriosis cells (Figure 4D,E).

### 2.4 In Vivo Magnetothermal Therapy in Endometriosis

**Figure 5:**
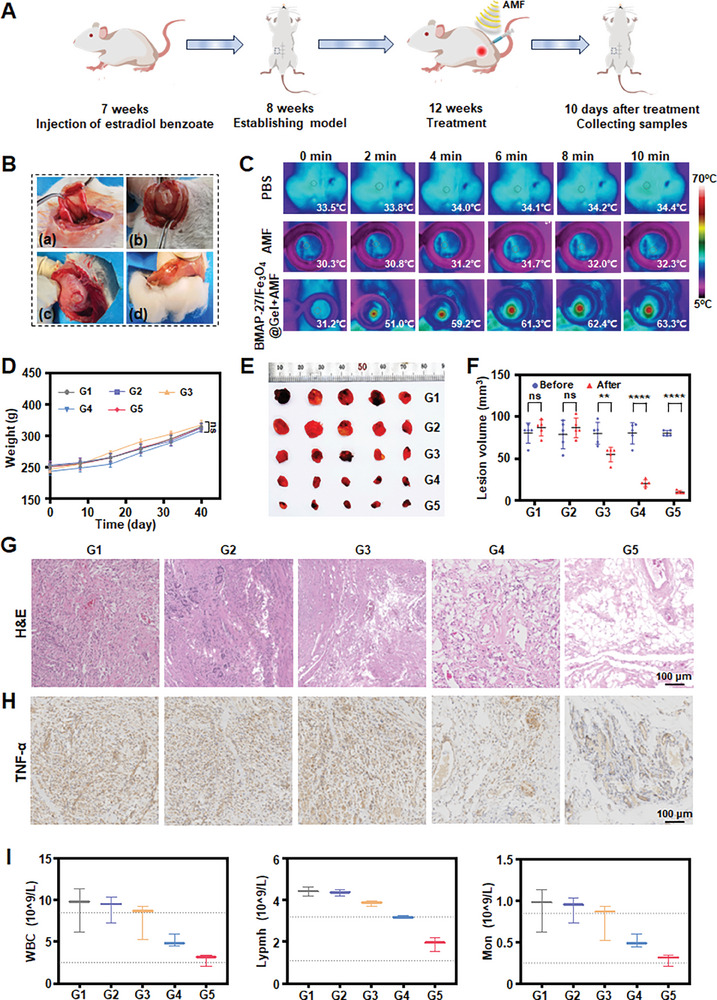
In vivo magnetothermal therapy in endometriosis. A) Schematic of the rat endometriosis treatment protocol. B) Establishment of the endometriosis model. C) Temperature variations at endometriotic lesion sites under different treatment conditions. D) Changes in rat body weight following various treatments (*n* = 5). E,F) Volumetric alterations in endometriotic lesions before and after treatment (*n* = 5). G) Representative H&E staining images of endometriotic lesions after different treatments. Scale bar = 100 µm. H) Immunohistochemical staining for TNF‐α expression (brown) in endometriotic lesion tissues. Scale bar = 100 µm. I) Quantification of white blood cell, lymphocyte, and monocyte counts in the blood of SD rats across different groups (*n* = 3). G1: PBS; G2: AMF; G3: BMAP‐27/Fe_3_O_4_@Gel; G4: Fe_3_O_4_@Gel+AMF; G5: BMAP‐27/Fe_3_O_4_@Gel+AMF. **p < 0.01, ****p < 0.0001, and ns: no significance. Statistical significance was determined using one‐way ANOVA.

To confirm the efficacy of magnetothermal therapy in treating endometriosis, we conducted in vivo investigations, extending the insights gained from our previous in vitro experiments. The experimental scheme is illustrated in **Figure** **5**A. We used the autologous transplantation method, recognized as the most widely adopted approach, to create an endometriosis model in rats.^[^ ^25^ ^]^ Specifically, a section of the uterine body was ligated, excised, and sutured onto the right lower quadrant of the abdominal wall following estradiol benzoate injection (Figure 5B‐a,b). The rats were anesthetized, and open surgery was performed to facilitate direct observation of the abdominal cavity and bilateral uterine areas. Healthy uterine tissue was carefully removed and sutured to the right lower abdominal wall. Four weeks post‐surgery, tissue samples were harvested for histological analysis using H&E staining to confirm the successful establishment of the endometriosis model (Figure S12, Supporting Information).^[^ ^15b^ ^]^ Endometriotic lesions exceeding 4 mm in length were considered indicative of a successful model (Figure 5B‐c). Following the model's establishment, the rats were randomly assigned to five experimental groups: G1) PBS, G2) AMF, G3) BMAP‐27/Fe_3_O_4_@Gel, G4) Fe_3_O_4_@Gel+AMF, and G5) BMAP‐27/Fe_3_O_4_@Gel+AMF. Treatment commenced with the injection of hydrogel at the lesion site. To evaluate the in vivo magnetic heat conversion effect of the drug‐loaded hydrogel, the rats were subjected to an external AMF (400 kHz, 1.2 kA m^−1^) for 10 min, with real‐time temperature monitoring performed using a thermal imaging camera. The temporal changes in temperature at the lesion site were recorded (Figure 5C). The BMAP‐27/Fe_3_O_4_@Gel+AMF group exhibited a marked temperature increase from 31.2 °C to 63.3 °C, significantly surpassing the temperature changes observed in the PBS group (an increase of 0.9 °C) and the AMF group (an increase of 2 °C). These findings underscore the significant potential of BMAP‐27/Fe_3_O_4_@Gel in magnetothermal therapy.

Post‐treatment, the rats were monitored for 10 days to assess growth and evaluate treatment efficacy. As shown in Figure 5D, the body weights across all groups remained stable between the modeling and treatment phases. With the exception of a slight reduction in the BMAP‐27/Fe_3_O_4_@Gel group, Figure 5E,F demonstrates that the volume of endometriotic lesions remained largely unchanged in the PBS and AMF groups. Conversely, lesion volumes significantly decreased in the Fe_3_O_4_@Gel+AMF and BMAP‐27/Fe_3_O_4_@Gel+AMF groups, with the most pronounced reduction observed in the latter. Histopathological analysis of H&E‐stained lesions revealed considerable vacuolization in the Fe_3_O_4_@Gel+AMF and BMAP‐27/Fe_3_O_4_@Gel+AMF groups, indicating cell cycle arrest and diminished cell proliferation compared with the control and other treatment groups (Figure 5G).

**Figure 6:**
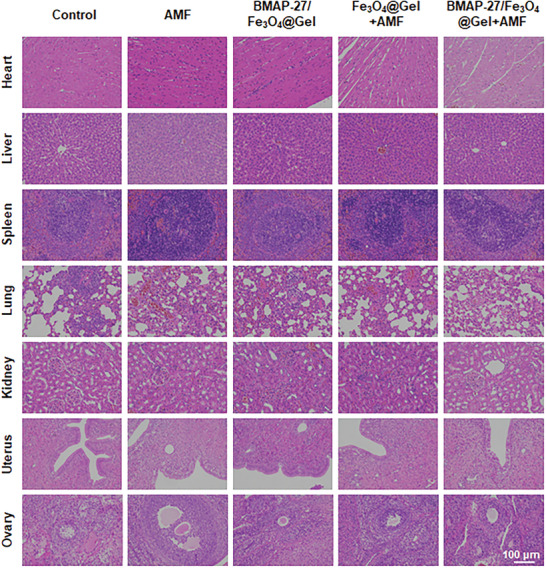
H&E staining of the heart, liver, spleen, lung, kidney, uterus, and ovary tissues from rats post‐treatment. Scale bar = 100 µm.

Additionally, systemic and local inflammation levels were assessed through peripheral blood tests and TNF‐α immunohistochemistry staining of ectopic lesion tissues. The BMAP‐27/Fe_3_O_4_@Gel+AMF group exhibited a significant reduction in both TNF‐α expression and white blood cell count (Figure 5H,I; Figure S13, Supporting Information). These results collectively demonstrate the therapeutic efficacy of BMAP‐27/Fe_3_O_4_@Gel under AMF influence in the treatment of endometriosis. Additionally, we quantified the accumulation of Fe_3_O_4_ NPs in the heart, liver, spleen, lung, kidney, and brain at various time points (0, 2, 5, 10 days) using inductively coupled plasma‐optical emission spectrometry (ICP‐OES). As shown in Figure S14 (Supporting Information), Fe_3_O_4_ NPs were retained in the spleen and liver, but not in the heart or brain.^[^ ^26^ ^]^ To assess in vivo degradation, BMAP‐27/Fe_3_O_4_@Gel was injected into the abdominal region of mice, with samples collected on days 0 and 10. Notably, after 10 days, BMAP‐27/Fe_3_O_4_@Gel exhibited noticeable degradation and fragmentation (Figure S15, Supporting Information). Importantly, this treatment demonstrated excellent biocompatibility, as no appreciable adverse effects were observed in the rats’ organs (**Figure** **6**).

## 3 Conclusion

In summary, we have developed a novel therapeutic strategy for endometriosis using AMF‐mediated magnetothermal effects. The synthesized injectable hydrogel, BMAP‐27/Fe_3_O_4_@Gel, demonstrated robust magnetothermal properties, facilitating the gradual softening of the hydrogel and subsequent release of the antimicrobial peptide BMAP‐27. Experimental results showed that the local lesion temperature in the BMAP‐27/Fe_3_O_4_@Gel group rapidly reached 63.3 °C, effectively ablating ectopic endometrial cells through non‐invasive hyperthermia. Furthermore, this treatment prompted the rapid release of BMAP‐27, which contributed to the modulation of the inflammatory microenvironment. This was evidenced by a significant reduction in TNF‐α levels within endometriosis lesions post‐treatment, thereby synergistically inhibiting the initiation and progression of endometriosis. Notably, biocompatibility and safety assessments, including histological analysis via H&E staining and body weight monitoring of experimental rats, demonstrated no significant toxicity associated with BMAP‐27/Fe_3_O_4_@Gel in major organs. Therefore, this AMF‐responsive injectable BMAP‐27/Fe_3_O_4_@Gel nanosystem holds substantial promise as a therapeutic option for endometriosis.

## 4 Experimental Section

### Synthesis of Fe_3_O_4_ and BMAP‐27/Fe_3_O_4_@Gel

The Fe(acac)_2_ (1 g) was dissolved in a mixture of ethylene glycol (30 mL) and diethylene glycol (20 mL) and stirred at 80 °C for 30 minutes. Then, Sodium acrylate (1.5 g) was added to the mixture and stirred for another 30 min. After that, sodium acetate (2 g) was added, and the mixture was continued to be stirred for 45 min to form a transparent burgundy solution. The solution was transferred to a stainless steel autoclave lined with polytetrafluoroethylene and kept at 200 °C for 24 h. Finally, the black product was collected by washing at least three times with distilled water and anhydrous ethanol, followed by centrifugation.^[^ ^18a^ ^]^

A 0.25% agarose (AG) solution was heated to 100 °C for uniform dispersion for synthesizing BMAP‐27/Fe_3_O_4_@Gel, then cooled to 50 °C. Fe_3_O_4_ and BMAP‐27 peptide (2 mg mL^−1^) were dispersed in the 0.25% AG solution and quickly mixed in a volume ratio of 1:1:8. After cooling to room temperature, BMAP‐27/Fe_3_O_4_@Gels were formed.

### Characterization of Fe_3_O_4_ and BMAP‐27/Fe_3_O_4_@Gel

The microstructure and morphology of Fe_3_O_4_ were observed using transmission electron microscopy (HITACHI HT7800, Japan). Crystal structure measurements were conducted on an X‐ray diffractometer (MiniFlex 600, Rigaku, Japan). The magnetic hysteresis curve of Fe_3_O_4_ was tested using a vibrating sample magnetometer (LakeShore 8600, USA). Particle size distribution and zeta potential were measured using a dynamic light scattering detector (Nanobrook 90 Plus Pals, Brookhaven, NY, USA). Fourier transform infrared spectroscopy (Nicolet iS5, Thermo) was used to analyze Fe_3_O_4_ and BMAP‐27/Fe_3_O_4_@Gel. The rheological behavior of BMAP‐27/Fe_3_O_4_@Gel was tested in temperature scanning mode using a rheometer (HAAKE Mars 40, Thermo). The microstructure, morphology, and elemental distribution of BMAP‐27/Fe_3_O_4_@Gel and Gel were obtained using a scanning electron microscope (TESCAN MIRA LMS, Czech).

### Gelation Properties of BMAP‐27/Fe_3_O_4_@Gel

The freshly prepared BMAP‐27/Fe_3_O_4_@Gel (1 mL) was injected into the bottom of a vial and allow it to settle for 3 min. The vial was rotated 180 degrees and observe the hydrogel's flow.

### Injection Performance of the BMAP‐27/Fe_3_O_4_@Gel

The gel, Fe_3_O_4_@Gel, and BMAP‐27/Fe_3_O_4_@Gel were separately injected in a 1 mL plastic syringe through 3 different needles (20, 22, and 24 G). Use the pressure plate to press down the plunger, and the force on the plunger was recorded by the weighing sensor. All tests were performed on Material Test System (CMT, 6103, *n* = 3). At the same time, the freshly prepared BMAP‐27/Fe_3_O_4_@Gel (1 mL) was injected into a mold with a needle size of 22 G, let it sit for 5 min, then removed the mold and checked the injectability of the hydrogel.

### Rheological Analysis

All samples’ rheological tests were performed with a Discovery DHR‐3 rheometer (HAAKE Mars 40, Thermo) using a parallel plate. To measure the storage modulus (G′) and loss modulus (G″), oscillatory frequency sweeps were conducted at a frequency ranging from 0.01 to 10 Hz under a fixed strain of 1%, and oscillatory strain sweeps were conducted at a strain ranging from 0.01 to 100% under a fixed frequency of 1 Hz.

### Magnetic Thermodynamic Performance Testing

All magnetothermal experiments were conducted using a medium‐frequency induction heating system (Shuang ping SPG‐06‐II, China), with temperature data recorded by an infrared thermal imaging camera (FLIR C3 X). The test tubes were placed at the center of a water‐cooled magnetic induction copper coil. The parameters of the machine and samples were as follows: frequency (f) = 400 kHz, magnetic field strength = 1.2 kA m^−1^, solution volume = 1 mL, Fe mass (m_Fe_) = 1.859 mg. The heating capacity of Fe_3_O_4_@PAA nanoparticles under an alternating magnetic field (AMF) was quantified by specific absorption rate (SAR = C_w_ (dT/dt) (m_s_/m_Fe_)), where C_w_ is the specific heat capacity of water (4.18 kJ kg^−1^ K^−1^). To avoid potential errors, d_T_/d_t_ was calculated as the slope of the initial heating curve within the first minute. M_s_ is the mass of the colloidal solution, and m_Fe_ is the mass of iron elements in suspension. The samples of different Fe_3_O_4_ concentrations were subjected to the AMF for 10 min to validate the magnetothermal performance of Fe_3_O_4_, with temperature recorded every 30 s. Magnetothermal cycling experiments (4 cycles of AMF on/off) were conducted to evaluate the magnetothermal stability of Fe_3_O_4_ and BMAP‐27/Fe_3_O_4_@Gel. The sample was allowed to cool freely to room temperature after each cycle of 10 min with AMF irradiation (at 3 mg mL^−1^), and the temperature change of BMAP‐27/Fe_3_O_4_@Gel was measured using the same method.

### Dynamic Volume Changes of BMAP‐27/Fe_3_O_4_@Gel

The hydrogel was placed in an AMF (f = 400 kHz, H = 1.2 kA m^−1^), and photo was taken every 2 min. The dynamic volume changes of BMAP‐27/Fe_3_O_4_@Gel were calculated using the formula: V_t_/V_0_ = (d_t_/d_0_)3 (d_t_ was the diameter of the hydrogel at a specific time, and d_0_ was the initial diameter of the hydrogel).

### Cell Culture

All patients involved in the experiment signed informed consent forms allowing the use of their samples in the study. This study was approved by the Ethics Committee of Shandong Provincial Hospital affiliated to Shandong First Medical University. Endometrial tissues from patients with endometriosis were collected under sterile conditions, washed thoroughly with PBS, and homogenized. The samples were digested at 37 °C for 40 min with stirring every 10 min. The suspension was filtered through 154 and 40 mm cell sieves. The obtained cells were then cultured in complete DMEM/F12 medium. Human endometrial stromal cells (ESCs) were identified using immunofluorescence.^[^ ^13a^ ^]^

### In Vitro MTT Assay of BMAP‐27/Fe_3_O_4_@Gel

ESCs (2 × 10^5^ cells) were seeded in small culture dishes and then divided into 5 groups to different treatments: NC, AMF, BMAP‐27/Fe_3_O_4_@Gel, Fe_3_O_4_@Gel+AMF, and BMAP‐27/Fe_3_O_4_@Gel+AMF. Cell toxicity was verified using the MTT assay. Primary ESCs (1 × 10^5^ cells) were seeded in confocal culture dishes and cultured for 24 h. Then, hydrogels were placed in the center of the wells for various treatments. After 6 h of continued culture, ESCs were co‐stained with Calcein AM/PI, and cellular fluorescence was detected using confocal laser scanning microscopy.

### Flow Cytometry Analysis

ESCs (2 × 10^5^ cells) were seeded in small culture dishes and cultured for 24 h. The existing medium was replaced fresh culture medium, and hydrogels were placed in the center of the wells for different treatments. After 6 h of continued culture, ESCs were stained with fluorescein isothiocyanate (FITC) and 7‐aminoactinomycin D (7‐AAD), and the apoptosis was analyzed using an acoustic focusing flow cytometer (ATTUNE NXT).

### Ethical Statement

All animal studies were conducted in accordance with the guidelines approved by the Experimental Animal Ethics Committee of Shandong First Medical University Affiliated Provincial Hospital (NO. HSRF2023‐0037), and performed according to the National Institutes of Health Guide for the Care and Use of Laboratory Animals (1996). Female SD rats (weighing ≈200 g ± 10 g and aged 7 weeks) were purchased from Jinan Peng Yue Experimental Animal Breeding Co. Ltd. (Jinan, China).

### Treatment of Endometriosis in Vivo

The principle of the model was primarily based on the auto‐transplantation theory of endometriosis implants. Twenty‐five female rats were housed in a specific pathogen‐free environment. The animals had ad libitum access to food and water. Injection of estradiol benzoate (0.1 mL, 2 mg mL^−1^) into rats induces estrus and endometrial proliferation, making it easier to separate the endometrial layer from the muscle layer of the rat uterus. The estrous cycle of the rats was determined using vaginal smears. Rats in estrus were anesthetized with pentobarbital sodium and safely placed on the operating table. After evenly applying povidone‐iodine to the abdomen, an incision was made to locate the uterus. Subsequently, the uterus's sides and related blood vessels were ligated with sterile absorbable suture, leaving a one‐centimeter gap in the middle. A 1 cm tissue slice was excised with scissors and transferred to a sterile culture dish containing physiological saline. The endometrium was carefully extracted with sterile microscope forceps and divided into 5 × 5 mm fragments. Then these fragments were fixed on the abdominal wall. The incision was meticulously sutured, and iodine was applied. After inducing endometriosis, the rats underwent 4 weeks recovery period during which no drug intervention was administered. A criterion for successful model establishment is that the length of the ectopic tissue exceeds 4 mm, while lengths smaller than this indicate an unsuccessful model. Subsequently, the cohort of 25 rats was randomly divided into 5 groups: PBS, AMF, BMAP‐27/Fe_3_O_4_@Gel, Fe_3_O_4_@Gel+AMF, and BMAP‐27/ Fe_3_O_4_@Gel+AMF. After anesthesia with pentobarbital sodium, each rat underwent abdominal incision, and a single dose was administered by direct injection into the endometrial implants (500 µL). The incision was then sutured, treated with iodine. After 10 d of treatment, blood samples were collected from the rats, followed by euthanasia. Subsequently, the volume of the treated ectopic endometrial tissue was measured, and samples of ectopic uterine tissue were obtained. After the dewaxing and dehydration processes, the internal morphology of the ectopic endometrium were evaluated using H&E. Analysis of inflammatory levels in ectopic tissue using TNF‐α immunostaining. A digital pathology slide scanning system (MBMbio‐intelligence‐400) was used to capture image information of stained sections, and IHC scoring was performed using ImageJ.

### In Vivo Biodistribution and Biosafety Assessment

Tissues (heart, liver, spleen, lung, kidney, and brain) were collected and weighed. For biodistribution of Fe_3_O_4_ NPs, the samples were mixed with the digesting solution (HCl+HNO_3_, volume ratio 3:1) and heated at 80 °C. The iron content in the samples was measured by ICP‐OES (G8018A, Agilent, USA). In addition, rats were euthanized after treatment, and major organs (heart, liver, spleen, lungs, kidneys, uterus, and ovaries) were collected for histopathological analysis of major organs. The tissue samples were stained with H&E.

### Statistical Analysis

The GraphPad Prism 8.0 and Origin 2018 software were used to perform the statistical analysis. All experiments were performed in triplicate with independent tests conducted at least three times. All data were expressed as mean ± standard deviation (SD). Sample size (n) for each statistical analysis was added in Figure legends. One‐way analysis of variance (ANOVA) was used to analyze the statistical significance. A value of *p < 0.05, **p < 0.01, ***p < 0.001, and ****p < 0.0001 were considered to be statistically significant and highly significant respectively (n.s. = no significance).

## Conflict of Interest

The authors declare no conflict of interest.

## Supporting information



Supporting Information

## Data Availability

The data that support the findings of this study are available from the corresponding author upon reasonable request.;
