# Supplementary material for: Injectable Magnetic Hydrogel Incorporated with Anti‐Inflammatory Peptide for Efficient Magnetothermal Treatment of Endometriosis
Source: Adv Sci (Weinh). 2024 Oct 7;11(44):2409778. doi: 10.1002/advs.202409778 (PMC11600196; doi:10.1002/advs.202409778)
Supplement: Supplementary file 1 — Supporting Information [file ADVS-11-2409778-s001.docx]

Supporting Information

**Injectable Magnetic Hydrogel Incorporated with Anti-inflammatory Peptide for Efficient Magnetothermal Treatment of Endometriosis**

Huaichao Liu, Xiaohui Dai, Na Li, Le Zhang, Zihan Wang, Ke Ren, Yulei Li,* Xiao Sun,* and Jipeng Wan*

H. Liu, N. Li, L. Zhang, Z. Wang, Prof. Y. Li, Prof. X. Sun, Prof. J. Wan

Department of Gynecology

Ji Nan Key Laboratory of Diagnosis and Treatment of Major Gynaecological Disease

Shandong Provincial Hospital Affiliated to Shandong First Medical University

Jinan 250021, China

E-mail: [liyulei@sdfmu.edu.cn](mailto:sunxiao@sdfmu.edu.cn), [sunxiao@sdfmu.edu.cn](mailto:sunxiao@sdfmu.edu.cn), [wanjipeng@sdfmu.edu.cn](mailto:wanjipeng@sdfmu.edu.cn)

K. Ren, Prof. X. Dai

School of Chemistry and Pharmaceutical Engineering

Medical Science and Technology Innovation Center

Shandong First Medical University and Shandong Academy of Medical Sciences

Jinan 250000, China

**Materials and Methods**

**Materials**

Fe(acac)_2_ and bisphenylimide H33342 trihydrochloride (Hochest 33342) were obtained from Aladdin Chemical Co. Ltd. (Shanghai, China). Sodium acrylate was purchased from Bide Pharmaceutical Technology Co. Ltd. (Shanghai, China). Agarose (AG) was produced by ABCONE Co. Ltd. (Shanghai, China). Annexin V-FITC and 7-AAD apoptosis detection kits were supplied by Dalian Meilun Biotechnology Co. Ltd. (Dalian, China). Calcein AM/PI dye was sourced from Biotool Biotechnology Co. Ltd. (Shanghai, China). Sodium acetate, ethylene glycol, diethylene glycol, anhydrous ethanol, and dimethyl sulfoxide were obtained from Sinopharm Chemical Reagent Co. Ltd. (Shanghai, China). All chemical reagents were used as received without further purification.

**The BMAP-27 and Fe_3_O_4_ Delease Behavior in BMAP-27/Fe_3_O_4_@Gel**

BMAP-27/Fe_3_O_4_@Gel was incubated with PBS and subjected by external AMF (f = 400 kHz, H = 1.2 kA·m^-1^) or without AMF. The release of BMAP-27 from the BMAP-27/Fe_3_O_4_@Gel at different time points was detected by UV-vis spectroscopy. The release of Fe_3_O_4_ from the BMAP-27/Fe_3_O_4_@Gel at different time points was detected by ICP-OES (G8018A, Agilent, USA).

**The Controllable Degradation of BMAP-27/Fe_3_O_4_@Gel**

The degradation behavior of BMAP-27/Fe_3_O_4_@Gel was evaluated based on the degradation rate. BMAP-27/Fe_3_O_4_@Gel was externally irradiated using a medium-frequency induction heater (f = 400 kHz, H = 1.2 kA·m^-1^). The remaining hydrogel weight was measured every 12 h, and the degradation rate was calculated as follows: Degradation rate (%) = (W_t_/W_0_) × 100% (W_t_ is the weight of remaining hydrogel at different time intervals, and W_0_ is the initial weight of the hydrogel).

**Supporting Figures**

**
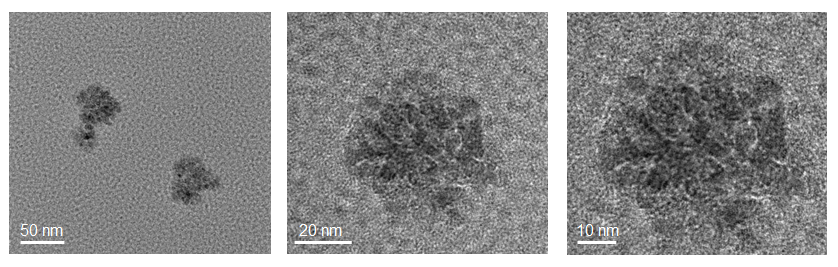
**

**Figure S1**. High magnification TEM image of Fe_3_O_4_.

**
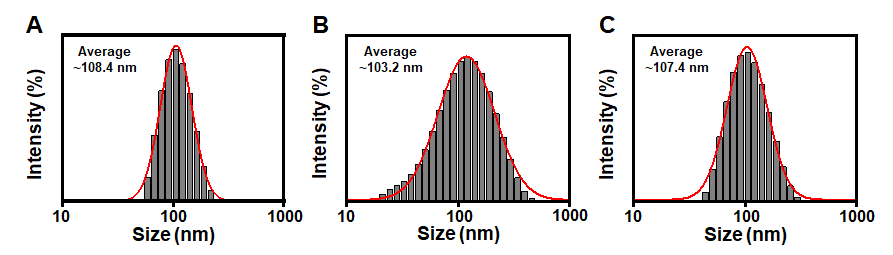
**

**Figure S2.** (A) DLS of Fe_3_O_4_ in PBS at 0 day (pH=7.4). (B) DLS of Fe_3_O_4_ in water at 14 days. (C) DLS of Fe_3_O_4_ in PBS at 14 days (pH=7.4).

**
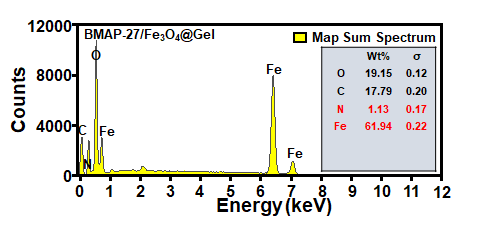
**

**Figure S3.** EDS analysis of BMAP-27/Fe_3_O_4_@Gel.


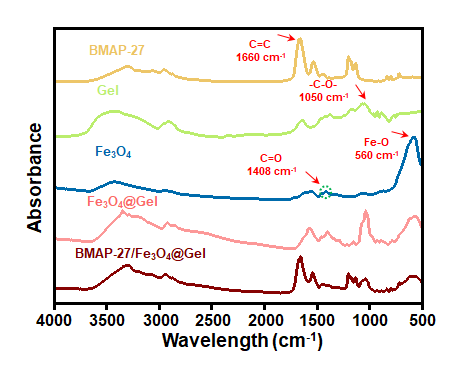


**Figure S4.** FTIR spectra of BMAP-27, AG, Fe_3_O_4_, Fe_3_O_4_@Gel, and BMAP-27/Fe_3_O_4_@Gel.


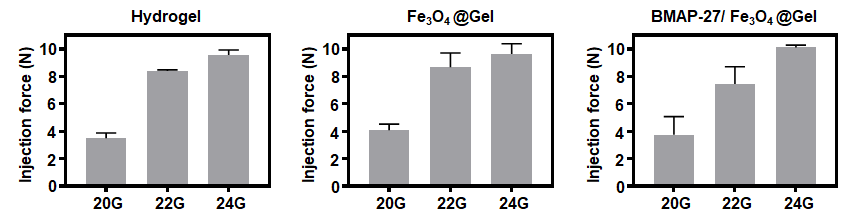


**Figure S5.** The injection force performance of hydrogel, Fe_3_O_4_@Gel, and BMAP-27/Fe_3_O_4_@Gel through three types of needles (20 G, 22 G, 24 G), n = 3.


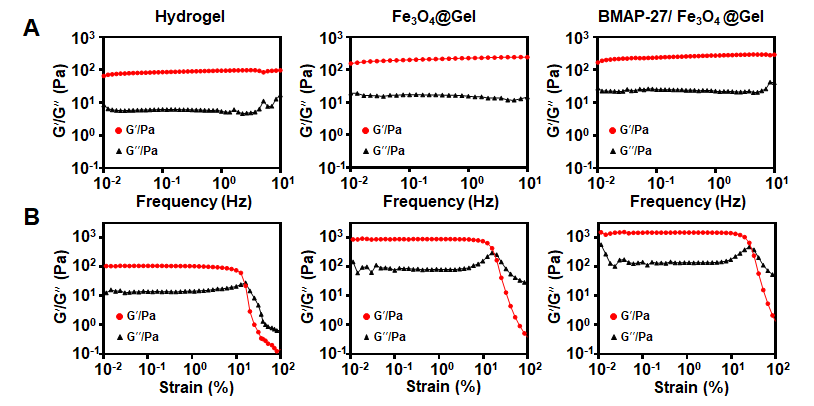


**Figure S6.** Rheological property of hydrogels. (A) Frequency sweeping of the storage (G′) and loss (G″) modulus (0.01-10 Hz, 1% strain). (B) Strain sweeping of the storage (G′) and loss (G″) modulus (0.01–100% strain, 1 Hz).


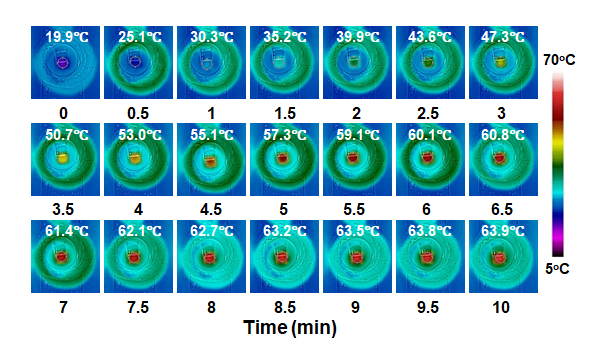


**Figure S7.** IR images of the BMAP-27/Fe_3_O_4_@Gel under magnetic hyperthermia in 10 minutes.

.


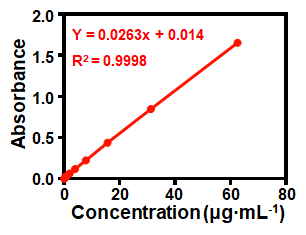


**Figure S8.** The standard curve of BMAP-27.

**
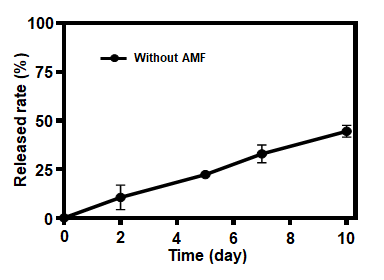
**

**Figure S9.** Released rate of Fe_3_O_4_ from BMAP-27/Fe_3_O_4_@Gel without AMF (n = 3).


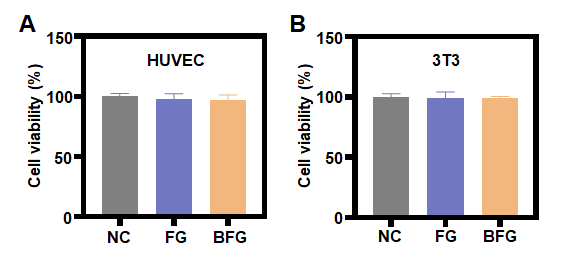


**Figure S10.** (A) Cell viability of HUVEC cells treated with Fe_3_O_4_@Gel (FG) and BMAP-27/Fe_3_O_4_@Gel (BFG) for 24 h (n = 5). (B) Cell viability of 3T3 cells treated with Fe_3_O_4_@Gel (FG) and BMAP-27/Fe_3_O_4_@Gel (BFG) for 24 h (n = 5).

**
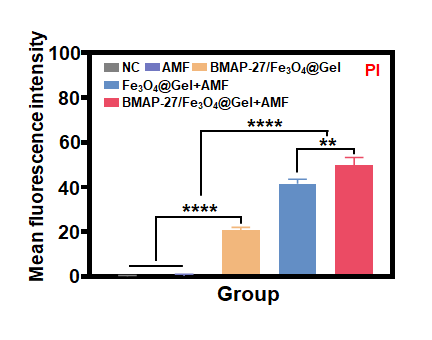
**

**Figure S11**. Quantitative analysis of PI. The data are presented as the mean ± standard deviation (n = 3). ** *p* < 0.01, **** *p* < 0.0001. Statistical significance was determined using one-way ANOVA.


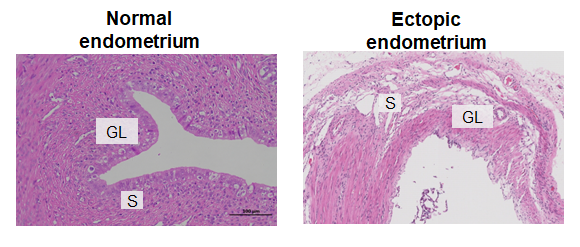


**Figure S12.** H&E of normal endometrium and ectopic endometrium in rat EMs models. GL represents glands, and S represents endometrial-like stroma.

**
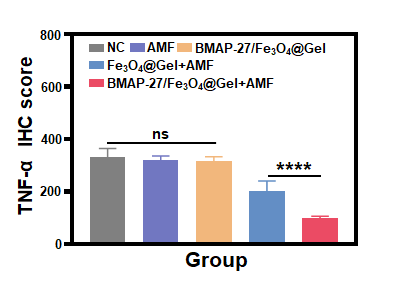
**

**Figure S13.** Quantitative analysis of TNF-α immunohistochemistry. The data are presented as the mean ± standard deviation (n = 3). **** *p* < 0.0001, and ns: no significance. Statistical significance was determined using one-way ANOVA.

**
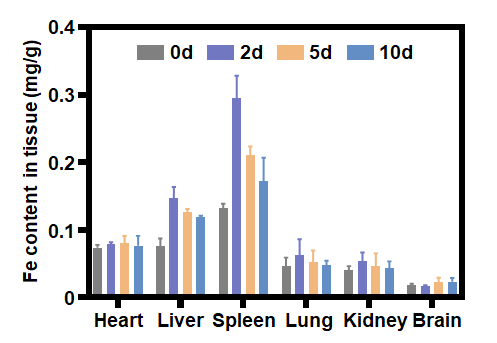
**

**Figure S14.** The tissues biodistribution of Fe_3_O_4_ NPs at 0 d, 2 d, 5 d and 10 days (n = 3)..


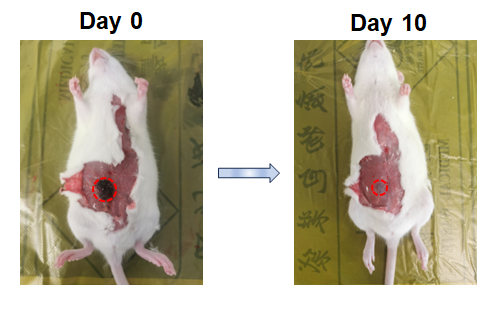


**Figure S15.** Assessment of BMAP-27/Fe_3_O_4_@Gel *in vivo* degradation at 0 day and 10 days.

**Table S1.** The dimension of clinical syringes needles used for testing injection force.

| **Needle** | **Length (mm)** | **Inner diameter (mm)** |
| --- | --- | --- |
| 20G | 160 | 0.6 |
| 22G | 160 | 0.4 |
| 24G | 160 | 0.3 |
